# Complete mitochondrial genome of the marine polychaete *Hediste japonica* (Phyllodocida, Nereididae)

**DOI:** 10.1080/23802359.2020.1717388

**Published:** 2020-01-24

**Authors:** Hyoung Sook Park, Sang-Eun Nam, Jae-Sung Rhee

**Affiliations:** a Department of Marine Science, College of Natural Sciences, Incheon National University, Incheon, South Korea;; b Research Institute of Basic Sciences, Incheon National University, Incheon, South Korea

**Keywords:** Polychaete, Hediste, *Hediste japonica*, mitogenome

## Abstract

To date, only five species are registered in the genus *Hediste*, and complete mitochondrial genome is reported in one species, *Hediste diadroma*. In this study, a complete 15,783 bp genome for the marine polychaete *H. japonica* mitochondrion was assembled through Illumina HiSeq platform. The complete mitochondrial genome of *H. japonica* contained 13 protein-coding genes (PCGs), 22 transfer RNA (tRNA) genes, two ribosomal RNA (rRNA) genes, and one control region. Overall genomic structure and gene orientation of *H. japonica* mitogenome are identical to those of *H. diadroma*. Phylogenetic analysis using the maximum likelihood method validated the sister relationship between *Hediste* sp. and other polychaetes. This information will be useful to understand geographical distribution, phylogenetic relationship, and evolutionary history of marine polychates.

Annelid is one of the largest metazoan with enormous complex diversity and is traditionally divided into Polychaeta and Clitellata as sister groups (Rouse and Fauchald 1995; Struck et al. 2011). Polychaetes are a very diverse group of segmented worms and are found in almost exclusively marine habitats (Nygren 2014). The family Nereididae comprises at least 540 species and 43 genera (Bakken and Wilson 2005). The genus *Hediste* (Malmgren 1867) is known to have five species and occupies a wide range of geographical distribution, ranging from the East Asia to the Atlantic and Pacific coastal regions (Smith 1958; Sato and Nakashima 2003). Although the complete mitochondrial genome of *H. diadroma* was firstly reported in the genus *Hediste* (Kim et al. 2016), research on the phylogenetic relationship and evolutionary history of the marine polychaete remains to be still explored due to their enormous morphological diversity, a wide range of ecological distribution and limited geographic samples, and insufficient genomic information.

In this study, we sequenced the complete mitogenome of *H. japonica* (Accession no. MN876864). An individual of *H. japonica* was isolated at the Ganghwa Mudflat Center (Yeocha-ri, Hwado-myeon, Ganghwa-gun, Incheon, South Korea; 37°36′31.1″N 126°22′57.0″E). The voucher specimen was deposited in the Research Institute of Basic Sciences of Incheon National University (Specimen ID: 201805-Polychaete014). Genomic DNA was isolate from the whole body of *H. japonica* with the DNeasy Blood and Tissue kit (Qiagen, Hilden, Germany). The genomic DNA was qualified and quantified using a Qubit 4 Fluorometer (Thermo Fisher Scientific, Inc., Waltham, MA, USA). The library construction and sequencing was performed by a commercial company (Macrogen, Seoul, South Korea). Genomic libraries were constructed from total genomic DNA (1 μg) using the TruSeq RNA Sample Preparation Kit according to the manufacturer’s instructions (Illumina, San Diego, CA, USA). The generated raw reads were pre‐processed and adapter sequences, low quality reads (sequences with > 50% bases with quality value ≤ 5), reads with > 10% of unknown bases, and ambiguous bases were totally removed to obtain high quality reads for assembly. To generate contigs, *de novo* assembly was performed using high quality reads by various k-mer using A5-pipeline. Additional PCR procedure was conducted to confirm the DNA sequence of COI, cytB, and control region. Overall sequences were annotated by using the MITOS web-based software (Bernt et al. 2013) and detailed annotation were conducted with NCBI-BLAST (http://blast.ncbi.nlm.nih.gov).

The complete mitogenome of *H. japonica* was 15,783 bp in length and contained the typical set of 13 PCGs, 22 tRNAs, two rRNAs, and one control region. The mitogenome was circular form, as the end of the contig was overlapped to the beginning region of the contig. The nucleotide composition of *H. japonica* mitogenome was heavily biased toward A + T nucleotides, accounting for 32% A, 19% C, 15% G, and 34% T. Overall gene contents and their orientations of *H. japonica* mitogenome were identical to those of *H. diadroma* (Kim et al. 2016). Nucleotide similarity between the 13 PCGs of *H. japonica* and *H. diadroma* was approximately 84%. Phylogenetic distance was estimated using the concatenated set of whole 13 PCGs of *H. japonica* mitogenome with including of 9 published mitogenomes from Nereididae and 11 registered other worms (Figure 1). The phylogenetic analysis was performed using the maximum likelihood method, GTR + G + I model with a bootstrap of 1000 replicates. The *H. japonica* mitogenome was closely clustered with *H. diadroma* and is supported as sister taxa within Nereididae. In conclusion, the complete *H. japonica* mitogenome will provide valuable information to understand phylogenetic distance between marine polychaetes and their evolutionary history, as phylogenetic relationship and monophyly versus paraphyly of Polychaeta are still unsolved questions in annelid subtaxa (Bartolomaeus et al. 2005; Struck et al. 2011).

**Figure 1.**
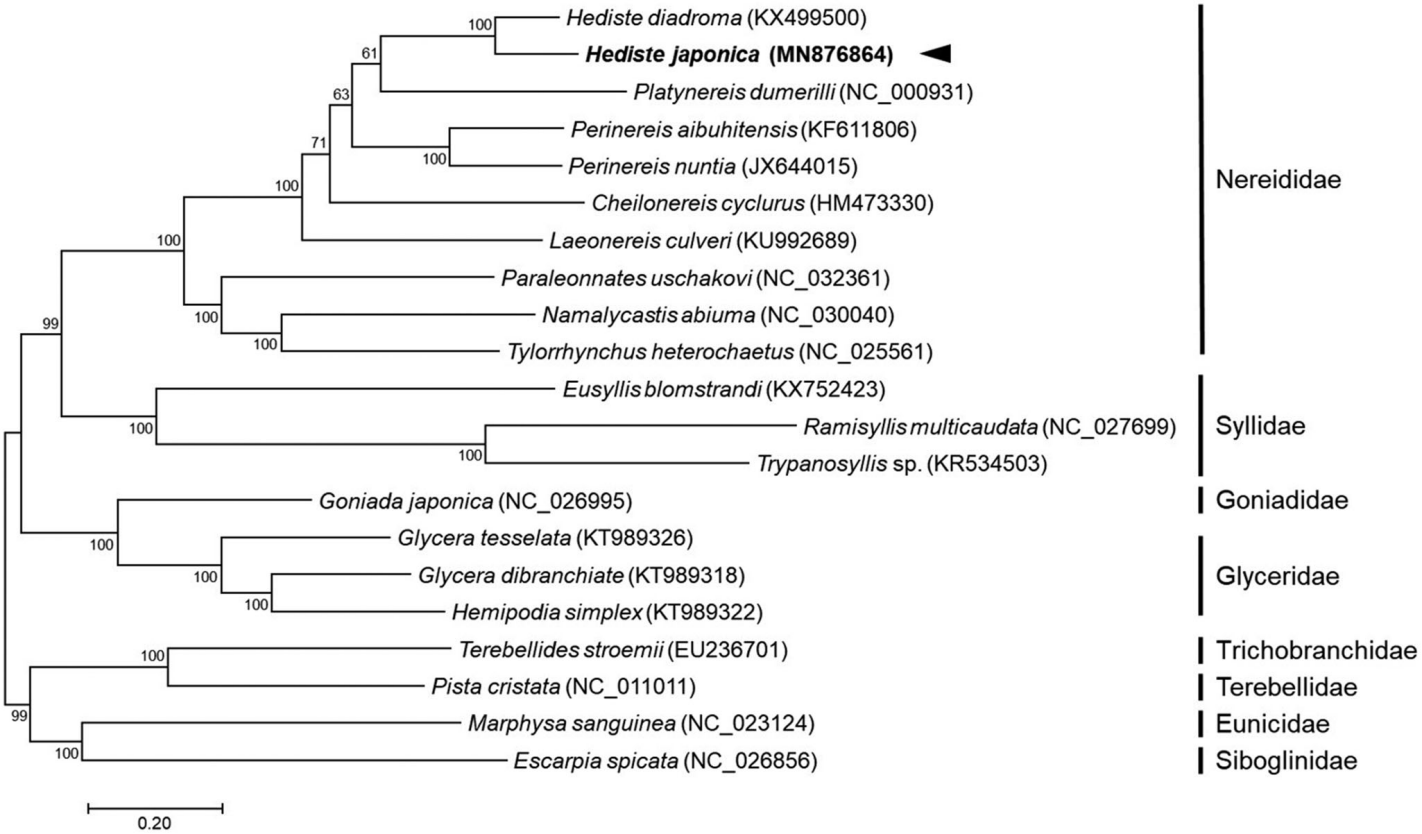
Maximum-likelihood (ML) phylogeny of 9 published mitogenomes from Nereididae and 11 registered other worms based on the concatenated nucleotide sequences of protein-coding genes (PCGs). Numbers on the branches indicate ML bootstrap percentages (1000 replicates). DDBJ/EMBL/Genbank accession numbers for published sequences are incorporated. The black arrow means the marine polychaete analyzed in this study.
